# Editorial: Novel Approaches in Cardiovascular Imaging: Case Reports

**DOI:** 10.3389/fcvm.2022.932990

**Published:** 2022-06-02

**Authors:** Antonios Karanasos, Grigorios Korosoglou

**Affiliations:** ^1^First Department of Cardiology, Athens Medical School, Hippokration Hospital, Athens, Greece; ^2^Department of Cardiology, Vascular Medicine and Pneumology, GRN Hospital Weinheim, Weinheim, Germany; ^3^Cardiac Imaging Center Weinheim, Hector Foundation, Weinheim, Germany

**Keywords:** cardiac magnetic resonance (CMR), cardiac computed tomography (CCT), multimodal imaging, fractional flow reserve (FFR), intravascular imaging

Continuous technical developments in multiple cardiovascular imaging modalities led to more profound and complete understanding of cardiac pathophysiology. These developments together with the wider availability of cardiovascular imaging may not only provide early and more precise diagnosis of cardiac disorders but also aid the development of novel treatment strategies, reducing the global burden of cardiovascular disease.

Due to its high practicability and bedside use, echocardiography is the first-line diagnostic tool for the assessment of cardiac dimensions and function. However, its capabilities to provide tissue characterization are limited. In this regard, the versatility of cardiac magnetic resonance (CMR) can provide assessment of myocardial function, perfusion, and viability all without ionizing radiation (1). Cardiac computed tomography (CCT) on the other hand, allows for the non-invasive assessment of coronary artery disease (CAD), assessing not only lumen narrowing but also its functional significance by non-invasive fractional flow reserve (FFR) (2). Due to its excellent spatial and high temporal resolution, CCT can also offer information related to the anatomical localization of cardiac masses and their relationship to the surrounding environment (3). In addition, CCT emerged as an important modality in the pre-procedural evaluation of the aortic valve and anulus metrics and morphology prior to transfemoral aortic valve implantation (TAVI) (4).

Case reports highlight unique cases of patients that present with an unexpected or unusual diagnosis, representing a valuable educational tool for clinicians. Within our special issue we aimed to highlight the importance of cardiovascular imaging for providing insights in terms of differential diagnosis, decision making, and clinical management in such cases.

The role of multimodality imaging is highlighted in the article by Tadic et al., where a 55-year-old male presented in the emergency department due to acute coronary syndrome. Echocardiography revealed the presence of an irregular mobile structure (~1.5^*^2cm) floating in the aortic bulbus. CT angiography additionally indicated critical ischemia due to its localization near the ostium of the left coronary artery, thus prompting urgent treatment by cardiac surgery. Hereby, a papillary fibroelastoma, attached to the Valsalva sinus could be successfully removed. A similar condition, where a structural disorder compromised the coronary circulation by compression of the left main was demonstrated in the article of Ning et al. In this case, a large aneurysm of the sinus of Valsalva, already demonstrated by transthoracic echocardiography, was confirmed by CCT as the cause of the limiting symptoms of the patient. CCT helped planning surgical treatment and evaluating the postoperative result. These articles nicely demonstrate the importance of CCT as part of a multimodality imaging evaluation in patients where structural conditions interfere with the coronary circulation (5).

Multimodality imaging also plays an important role in procedural planning, both for surgical and interventional procedures. Liang et al. presented a case where multimodality imaging by transthoracic echocardiography, CCT, and CMR allowed the identification of a mass located in the right atrioventricular groove, causing compression of the right heart. Surgery on a beating heart was performed based on imaging findings, and the mass was intraoperatively identified as a hematoma. Spontaneous coronary rupture was suspected by the authors as a possible cause, considering its proximity to the right coronary artery. Similarly, with TAVI procedures, imaging plays a central role in procedural planning. In a case by Aldauig et al., CCT identified an anomalous course of the left circumflex artery, which arose from the right sinus of Valsalva. This finding guided a high implantation of a balloon-expandable valve, which was performed after catheter engagement and wiring of the left circumflex artery.

The role of CCT-derived FFR in the non-invasive evaluation of the hemodynamic significance of coronary lesions is underscored in the case series of Gajanan et al. Several coronary lesions of patients, who had undergone CCT-FFR assessment were assessed both by angiography-derived FFR and by invasive FFR. Agreement was high among modalities for lesions without excessive calcification or prior stent implantation. However, in cases with severely calcified plaques and stents the accuracy of CT-FFR was rather modest, which was attributed to blooming artifacts related to calcifications and stent struts or to motion artifacts.

The ability of CMR to assess perimyocarditis due to SARS-COVID vaccinations is highlighted by the articles by Korosoglou et al. and Ansari et al. Recently, several studies demonstrated acute myocarditis in previously healthy young individuals, particularly in males between 16-39 years after vaccination with mRNA SARS-CoV-2 vaccines (6–9). In the present case reports, two young male patients presented with fatigue, myalgia, and chest pain 2 days after the second SARS-CoV-2 vaccination with BNT162b2 and mRBA-1273, respectively (Ansari et al., Korosoglou et al.). These cases share similarities since CMR exhibited clear signs of acute myocardial inflammation with abundant non-CAD related late gadolinium enhancement (LGE), indicative of acute myocarditis, while favorable clinical courses were noticed and follow-up CMR examinations exhibited full functional recovery and disappearance of LGE. These cases highlight the role of CMR not only for the initial diagnosis but also for the follow-up of such patients, where the risk of relatively rare adverse effects due to vaccination, needs to be balanced against the benefits of protection from severe forms and complications due to the COVID-19 disease.

In the field of intravascular imaging, the case by Dhawan et al. showcases the utility of optical coherence tomography (OCT) to guide treatment in selected cases with ST-segment elevation myocardial infarction (STEMI). In selected cases of young patients with STEMI due to plaque erosion (10, 11), an intensified antithrombotic therapy may obviate stent implantation. Such a ‘leave nothing behind' strategy may bear potential long-term benefits.

In conclusion, recent advances with non-invasive and invasive imaging have contributed to precise diagnostic work-up of patients guiding timely and efficient treatment. The cases reported in the present article collection are excellent examples, showing how multimodality imaging can be incorporated in the daily clinical practice to improve patient care and outcomes.

## Author Contributions

Both authors have made a substantial, direct, and intellectual contribution to the work and approved it for publication.

## Conflict of Interest

The authors declare that the research was conducted in the absence of any commercial or financial relationships that could be construed as a potential conflict of interest.

## Publisher's Note

All claims expressed in this article are solely those of the authors and do not necessarily represent those of their affiliated organizations, or those of the publisher, the editors and the reviewers. Any product that may be evaluated in this article, or claim that may be made by its manufacturer, is not guaranteed or endorsed by the publisher.
